# Diagnostic and Prognostic Potential of Tetranectin in Heart Failure and Cardiovascular Disease: A Systematic Review

**DOI:** 10.3390/medsci13040206

**Published:** 2025-09-24

**Authors:** Paula Alexandra Vulciu, Luminita Pilat, Maria-Daniela Mot, Paula Irina Barata, Imola Donath Mikos, Mos Raluca Stefana Ioana, Alexandru Alexandru, Cristiana-Smaranda Ivan, Norberth-Istvan Varga, Narcisa Carmen Mladin, Maria Puschita

**Affiliations:** 1 Department of Biochemistry, “Vasile Goldis” Western University, B-dul Revolutiei Nr. 96, 310025 Arad, Romania; vulciu.paula@uvvg.ro (P.A.V.); luminita.pilat@yahoo.com (L.P.); 2 Department of General Medicine, “Vasile Goldis” Western University, B-dul Revolutiei Nr. 96, 310025 Arad, Romania; mt_dana@yahoo.com; 3 Department of Physiology, “Vasile Goldis” Western University, B-dul Revolutiei Nr. 96, 310025 Arad, Romania; barata_paula@yahoo.com (P.I.B.); miklos.imola@uvvg.ro (I.D.M.); 4 Department of Cardiology, “Vasile Goldis” Western University, B-dul Revolutiei Nr. 96, 310025 Arad, Romania; mos.raluca@uvvg.ro; 5 Department of General Medicine, “Victor Babeş” University of Medicine and Pharmacy, Eftimie Murgu Square, No. 2, 300041 Timișoara, Romania; alexandru.alexandru@student.umft.ro (A.A.); smaranda.ivan@student.umft.ro (C.-S.I.); 6 Doctoral School, Department of General Medicine, “Victor Babeş” University of Medicine and Pharmacy, Eftimie Murgu Square, No. 2, 300041 Timișoara, Romania; 7 Department of Internal Medicine, “Vasile Goldis” Western University, B-dul Revolutiei Nr. 96, 310025 Arad, Romania; mladin_narcisa@yahoo.com (N.C.M.); mpuschita.mp@gmail.com (M.P.)

**Keywords:** tetranectin, CLEC3B, heart failure, coronary artery disease, cardiac biomarker, fibrosis, prognosis, NT-proBNP

## Abstract

Background: Tetranectin (CLEC3B), a plasminogen-binding protein involved in fibrinolysis and tissue remodeling, has been increasingly studied as a potential diagnostic and prognostic biomarker in cardiovascular disease (CVD). This review synthesizes current evidence on its clinical utility across heart failure (HF), coronary artery disease (CAD), and related conditions. Objectives: To systematically evaluate and synthesize published clinical evidence on the diagnostic and prognostic value of tetranectin in cardiovascular diseases. Methods: A systematic search of PubMed, Google Scholar, and Scopus (January 2010–June 2025) identified original human studies examining associations between tetranectin (CLEC3B) and cardiovascular diseases, including heart failure, coronary artery disease, myocardial infarction, and cardiometabolic conditions. Eligible studies included adult cohorts with observational designs; experimental, in vitro, and pediatric studies were excluded. Two reviewers independently extracted data on study design, population characteristics, biomarker assessment, and outcomes, resolving discrepancies by consensus. Results: Twelve studies were included. Tetranectin levels were consistently lower in patients with CAD, MI, and advanced HF compared to controls. Higher circulating TN levels were associated with reduced risk of HF onset, cardiovascular death, and hospitalization. In two studies, combining tetranectin with NT-proBNP improved diagnostic accuracy over NT-proBNP alone. Mechanistic studies revealed correlations between TN expression and fibrosis-related gene pathways, supporting its biological relevance. Conclusions: Tetranectin shows consistent promise as a diagnostic and prognostic biomarker in cardiovascular disease, particularly in heart failure and coronary artery disease. Its involvement in fibrotic remodeling, plasminogen activation, and vascular homeostasis underlines biological pathways relevance. Combining tetranectin with established biomarkers may improve cardiovascular risk stratification and guide more personalized therapeutic strategies. Further large-scale and longitudinal studies are needed to validate its clinical utility across diverse settings.

## 1. Introduction

Cardiovascular disease (CVD) remains the leading cause of death worldwide, with a growing burden in developing countries, main cause being the rapid epidemiological transition from infectious towards chronic diseases, over the past two decades [1,2,3]. This shift has brought prevention of CVD in foreground, with public health measures aiming to reduce CVD incidence. However, targeted primary preventive measures are better as they funnel resources on populations at higher risk. The present CVD risk evaluation methods demonstrate restricted predictive capability and may show poor calibration when implemented across different population groups [4].

The development of new diagnostic and therapeutic strategies faces challenges because CVDs possess intricate and multiple complex factors that contribute to their occurrence [5,6,7]. Multiple risk factors including obesity and diabetes mellitus, and hypertension combine through various biological mechanisms which makes both early detection and precise intervention more complicated [8,9]. In this context, the identification of novel biomarkers capable of improving diagnostic precision and guiding personalized therapy is essential for reducing CVD-related morbidity and mortality.

The diagnosis and management of cardiac events improved considerably through the use of traditional cardiac biomarkers including troponins and natriuretic peptides [10,11]. The current limitations of these biomarkers which affect their performance in early disease detection and chronic conditions demonstrate the necessity for additional molecular markers that detect various cardiovascular disease pathology [12,13].

Tetranectin (TN) also sometimes referred by its coding gene name CLEC3B (C-Type Lectin Domain Family 3, Member B), is a plasminogen-binding protein first identified in 1986, has emerged as a promising candidate biomarker due to its roles in tissue remodeling and systemic stress responses [14]. Tetranectin is a liver-derived, homotrimeric C-type lectin that binds plasminogen and enhances its activation, thereby promoting fibrinolysis and protecting against thrombotic burden [15]. It also regulates extracellular matrix remodeling and fibrosis through interactions with collagen-associated proteins, contributing to tissue repair, bone mineralization, and myogenesis [16,17,18]. Notably, early evidence from Wewer et al. (1998), demonstrated a specific and temporally regulated expression pattern of tetranectin during murine skeletal muscle development [16]. Tetranectin appeared in myoblasts and myotubes from embryonic day 12.5, peaked during myofiber maturation, and declined after birth, suggesting a role in myogenesis and cell–matrix interactions at junctional sites [16]. Tetranectin also reappeared during muscle regeneration after injury and was observed in differentiating and embryonic stem cells in vitro, further supporting its involvement in myofiber formation and muscle morphogenesis [16].

These findings laid the groundwork for later studies exploring tetranectin’s role in muscle biology, extracellular matrix dynamics, and tissue repair processes [19,20,21].

Originally recognized for enhancing plasminogen activation and promoting fibrinolysis, tetranectin has since been implicated in a broad spectrum of diseases, including cardiovascular disorders, diabetes, neurodegeneration, cancer, and sepsis [14,15,22,23,24,25].

In healthy individuals, serum tetranectin levels typically average around 10 mg/L but decline significantly in pathological states such as cancer and advanced CVD [14,15,26]. In cancer, for example, tetranectin accumulates in the extracellular matrix (ECM) at the invasive front, reflecting its involvement in structural remodeling [27]. The protein also plays key roles in developmental processes such as bone mineralization, muscle regeneration, and stem cell differentiation [15,17,28,29]. Moreover, tetranectin interacts with various proteins—including growth factors and transcriptional regulators—suggesting it may influence disease progression via altered cell signaling [15,18,30,31].

Within cardiovascular contexts, altered circulating tetranectin levels have been reported in coronary artery disease (CAD), myocardial infarction, and diabetes [26,32,33]. In CAD, serum levels were significantly lower than in healthy controls and declined progressively with disease severity [26]. These associations with inflammation and vascular remodeling suggest that tetranectin may reflect underlying pathological changes in the cardiovascular system.

Among cardiovascular conditions, heart failure (HF) appears particularly relevant. Multiple studies consistently report that higher tetranectin levels are associated with a lower risk of incident HF, cardiovascular mortality, and all-cause death [34,35,36]. Conversely, lower levels have been linked to adverse outcomes in conditions such as anthracycline-induced cardiotoxicity and advanced HF [37,38].

At the tissue level, tetranectin expression has shown strong correlations with fibrosis-related genes and myocardial collagen content, pointing to its role in cardiac remodeling [38].

This systematic review aims to evaluate the current clinical evidence regarding the role of tetranectin as a diagnostic and prognostic biomarker in cardiovascular diseases, including HF, myocardial infarction, and coronary artery disease. By synthesizing data across various clinical settings and disease stages, this review seeks to clarify the potential of tetranectin in improving risk stratification and guiding therapeutic interventions in cardiovascular care.

## 2. Materials and Methods

### 2.1. Guidelines and PICO

This systematic review followed the guidelines of the Preferred Reporting Items for Systematic Reviews and Meta-Analyses (PRISMA) 2020 statement [39]. This review has been prospectively registered in PROSPERO international systematic review registry (PROSPERO CRD420251126174).

The research question was structured according to the PICO framework, as follows:

- Participants: Patients > 18 years old with cardiovascular conditions, including HF, myocardial infarction, coronary artery disease or inflammatory cardiac diseases.
- Intervention/Index: Evaluation of TN levels in blood or tissue, either as a primary biomarker or as part of a proteomic panel.
- Comparison: Patients without cardiac disease, or those with varying levels of TN; in some studies, comparisons across disease stages, biomarker panels, or standard cardiac markers (e.g., NT-proBNP).
- Outcome: Clinical or molecular outcomes, including disease severity, functional cardiac parameters (e.g., ejection fraction), prognosis, mortality, fibrosis scores, and biomarker expression patterns.

### 2.2. Search Strategy

We conducted a systematic literature search using PubMed, Google Scholar, and Scopus. This multi-platform approach was selected to minimize the limitations inherent to each individual database, such as Boolean operator constraints in ScienceDirect and the broader, less-specific indexing in Google Scholar.

The search strategy combined Medical Subject Headings (MeSH) and free-text keywords, ensuring a broad and inclusive capture of relevant literature. Search terms included: “tetranectin”, “CLEC3B”, “cardiac biomarker”, “heart failure”, “myocardial infarction”, “cardiomyopathy”, “coronary artery disease”, “atherosclerosis”, “cardiac fibrosis”, “extracellular matrix”, “cardiac remodeling”, “proteomics”, and “cardiovascular disease”.

Boolean operators (AND, OR) were used to refine the results. An example of a typical search string was:

(“Tetranectin” OR “CLEC3B”) AND (“Heart Failure” OR “Myocardial Infarction” OR “Cardiomyopathy”) AND (“Biomarker” OR “Fibrosis” OR “Proteomics” OR “Prognosis” OR “Extracellular Matrix”).

The search was limited to articles published in English and included original human studies, proteomic investigations, and mechanistic studies relevant to tetranectin in the context of cardiovascular disease.

### 2.3. Selection of Articles

To ensure the thoroughness and reliability of the included sources, two reviewers independently assessed each publication for eligibility. Discrepancies in judgment were resolved through discussion, and when consensus could not be reached, a third reviewer was consulted. For the screening process, two independent reviewers (L.P. and P.I.B.) evaluated all records for eligibility. The inter-rater reliability, measured by Cohen’s Kappa, was 0.81, indicating a high level of agreement. Any remaining disagreements were resolved by reaching consensus or, if needed, by arbitration with a third reviewer (N-I.V.).

### 2.4. Inclusion Criteria

Articles were selected based on the following inclusion criteria:

(1) Participants ≥ 18 years old.
(2) Studies with abstracts relevant to the potential role of TN as a biomarker in cardiovascular diseases, including but not limited to heart failure, myocardial infarction, cardiomyopathy, coronary artery disease, or congenital/inflammatory cardiac conditions.
(3) Studies that report clinical or biological outcomes such as disease severity, prognosis, mortality, cardiac function (e.g., ejection fraction), fibrosis scores, or relevant biomarker levels.
(4) Full-text original research articles published in English between 1 January 2015 and 1 June 2025.
(5) Studies involving human participants or animal models, specifically observational cohort studies, case-control studies, or interventional trials, as well as mechanistic proteomic or genomic studies with clear cardiovascular relevance.

Findings from animal or in vitro studies were considered only for context within the Introduction or Discussion.

### 2.5. Exclusion Criteria

The exclusion criteria were:

(1) Participants < 18 years old.
(2) Studies not focused on cardiovascular disease or not assessing the relationship between TN and cardiac outcomes.
(3) Studies with fewer than 10 participants.
(4) Publications not appearing in peer-reviewed journals.
(5) Studies lacking published/accessible full-text (abstract-only).
(6) Publications with unsuitable formats, such as letters, case reports, editorials, conference abstracts, or systematic reviews.

Pediatric studies were excluded to maintain a more homogeneous population, as disease mechanisms and clinical responses in children may differ substantially from adults; however, relevant pediatric findings are discussed separately for context.

After finalizing the list of eligible studies, two independent reviewers (M.R.S.I and N.C.M.) systematically extracted relevant data using a pre-defined, standardized table. Extracted information included: study identifier (first author and year), study design, cohort size, patient characteristics, analytic methods, treatment details, and disease context and the main reported outcomes. Any missing or unclear information was noted and considered as limitation during the critical appraisal process.

Studies were selected for each synthesis based on the availability of the relevant outcome data. Given the variability in reporting formats and metrics across studies, no imputation methods were applied for missing data. All analyses were conducted based solely on the data available from the original publications.

### 2.6. Quality Assesment of the Studies

Two reviewers (L.P. and P.I.B.) independently evaluated the methodological quality of the included studies using the National Institutes of Health (NIH) Study Quality Assessment Tools (available at www.nhlbi.nih.gov/health-topics/study-quality-assessment-tools; accessed on 1 July 2025). Any disagreements between the reviewers were resolved through discussion, and when necessary, by consulting a third reviewer (N-I.V.). The results of the quality assessment are presented in Supplementary Material, Table S1. The results of the quality assessment are presented in Supplementary Material, Table S1. Overall, 10 of 12 studies were rated good and 2 fair; study designs were predominantly prospective cohorts (6/12), with three cross-sectional and three retrospective. The most frequent concerns were absent sample-size/power justification and reliance on single time-point tetranectin measurement.

## 3. Results

### 3.1. Overview of Included Articles

A total of 721 records were identified through database searches, including PubMed (*n* = 284), ScienceDirect (*n* = 392), and Google Scholar (*n* = 45). After removing 193 duplicate entries and 364 records through automated filters and screening tools (based on language, publication type, and relevance to tetranectin and cardiovascular conditions), 164 records were screened by title and abstract.

Of these, 133 were excluded for the following reasons: irrelevant population (*n* = 59), unrelated exposure or outcome (*n* = 56), and inappropriate study design (*n* = 20). This left 29 full-text articles assessed for eligibility. After excluding 19 more studies with irrelevant exposure or outcomes, 10 studies were ultimately included from the database search.

**Figure 1:**
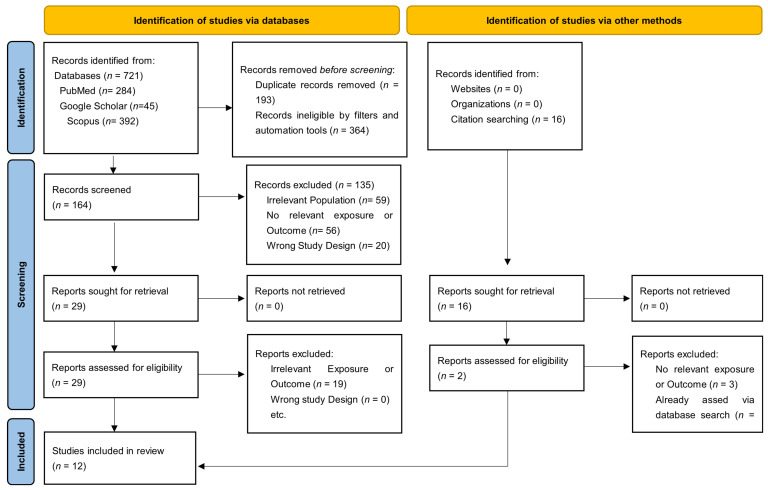
PRISMA Study Selection Workflow.

An additional 16 studies were identified via citation searching. Of these, 2 studies met the inclusion criteria after full-text review, while the rest were excluded due to prior inclusion (*n* = 11) or irrelevant outcomes (*n* = 3). The PRISMA workflow is illustrated in Figure 1.

This systematic review includes 12 studies investigating the role of TN as a cardiovascular biomarker across diverse patient populations and study designs. Sample sizes ranged from 10 to 18,383 participants, with most studies reporting a predominance of male patients (55–80%) and mean or median ages between 36.9 and 73.5 years. Study designs were primarily prospective cohorts (*n* = 6), with additional cross-sectional (*n* = 3) and retrospective or mixed-design studies (*n* = 2). Geographically, the research spans multiple countries, including the USA, China, Norway, Romania, and Australia.

Tetranectin was measured using ELISA kits, SomaScan assays, or LC-MS/MS-based proteomics. Outcomes varied across studies but focused largely on HF, myocardial infarction, coronary artery disease, cardiovascular mortality, and all-cause death. Several studies also explored associations with left ventricular function, myocardial fibrosis, or biomarker performance in risk stratification. Notably, higher tetranectin levels were consistently associated with reduced risk of HF, cardiovascular events, and mortality, while lower levels were linked to adverse outcomes, including anthracycline-related cardiac dysfunction.

Despite differences in methodology, these findings support tetranectin’s potential utility as a prognostic and diagnostic biomarker in cardiovascular disease. Table 1 summarizes the characteristics, methodologies, and key results of the included studies.

### 3.2. Tetranectin in Heart Failure and Myocardial Disfunction

Tetranectin has been consistently identified as a protective biomarker for HF in three large prospective cohort studies. Ho et al. (2018, USA, *n* = 3523) [34] first reported that higher circulating levels of tetranectin were significantly associated with reduced risks of incident HF (HR = 0.82), cardiovascular (CV) death (HR = 0.77), and all-cause mortality (HR = 0.82), with all *p*-values < 0.01. These findings were later confirmed in a larger proteogenomic cohort by Shah et al. (2024, USA and Norway, *n* = 18,383) [35], which reported nearly identical hazard ratios. A third study by Patel-Murray et al. (2024, USA, *n* = 1117) [36] focused specifically on HFpEF patients and found that higher tetranectin levels were associated with a reduced risk of HF hospitalization and CV death (adjusted rate ratio = 0.69; 95% CI: 0.51–0.94; *p* = 0.019). Together, these three studies provide strong and consistent evidence for tetranectin’s prognostic value across HF phenotypes.

One additional study explored tetranectin in comorbid or high-risk populations. Kopeva et al. (2023, Russia, *n* = 114) [37] found that serum tetranectin levels were significantly lower (by up to 33.7%) in patients with adverse outcomes from anthracycline-related cardiac dysfunction (ARCD), and levels continued to decline over 24 months. Tetranectin was an independent predictor of adverse events (OR = 7.08, *p* < 0.001), and predictive accuracy was markedly improved when combined with NT-proBNP (AUC = 0.954).

In a study by Dib et al. [41], TN was also assessed as one of the top proteins negatively associated with the composite endpoint of death or heart failure–related hospitalization (DHFA), as well as with all-cause mortality, with these findings replicated in the WashU Heart Failure Registry

In summary, five independent studies—including four large cohorts and two high-risk subpopulations—support tetranectin’s role as a biomarker for HF risk prediction and disease severity assessment.

All findings described above, can be found are summarized in Table 2 below.

### 3.3. Diagnostic Utility and Underlying Mechanisms of Tetranectin in Heart Failure

Beyond its prognostic value, TN has also been studied for its diagnostic accuracy and mechanistic involvement in myocardial remodeling. McDonald et al. [38] (*n* = 40) reported that plasma TN levels were significantly reduced in HF patients (*p* < 0.0001), with superior diagnostic accuracy compared to BNP (AUC = 0.97 vs. 0.84, *p* = 0.011). Furthermore, cardiac tissue expression of TN showed strong correlations with fibrosis-related markers including COL3A1, MMP9, TIMP1, and galectin-3 (all *p* < 0.05), as well as with histological collagen content (*r* = 0.55, *p* = 0.0019), suggesting a potential role in extracellular matrix remodeling [38]. Li et al. [42] (2024, Australia, *n* = 12) supported this with transcriptomic data showing significant upregulation of TN in dilated cardiomyopathy myocardial samples (FC = 3.1, adjusted *p* < 0.001). Similarly, Vulciu et al. (2025, Romania, *n* = 67) [43] identified tetranectin as an independent diagnostic mean of HF severity in patients with hypertension and dyslipidemia (OR = 0.998 per 1 mg/L increase; *p* = 0.002), even after adjusting for key confounders. Supporting these findings, Dib et al. [41] (*n* = 1167) found significant reductions in plasma TN levels in HF patients (*p* < 0.001).

On the other hand, a study by Dixit et al. [33] (*n* = 13) observed no significant changes in isolated HFpEF (fold change = 0.9). These contrasting findings between plasma and tissue analyses highlight a complex regulatory dynamic of TN with dependence of spatial situation, therefore the need for integrated multi-omics studies.

**Figure 2:**
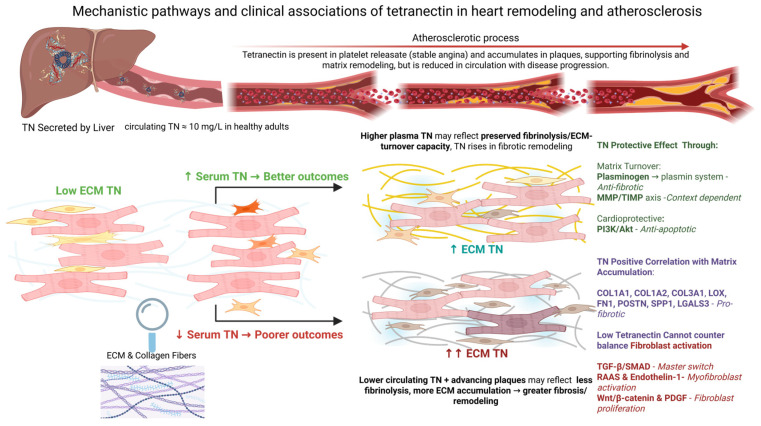
Mechanistic pathways and clinical associations of tetranectin in heart failure and atherosclerosis (All rights reserved).

These mechanistic pathways and their clinical implications are summarized schematically in Figure 2 (see also Section 4.3 for additional mechanistic insights and animal model data).

All findings described above, can also be found summarized in Table 3.

### 3.4. Context-Dependent Roles of Tetranectin Across Cardiovascular and Metabolic Diseases

Evidence links TN to both atherosclerotic disease progression and acute coronary events [26,32,40]. In a large cross-sectional study, Chen et al. [26] (*n* = 316) reported significantly lower serum TN levels in CAD patients compared to healthy controls (10.12 ± 3.41 mg/mL vs. 11.16 ± 3.17 mg/mL, *p* = 0.007). TN levels declined progressively with increasing disease severity, from CAD-negative to three-vessel disease (*p* for trend = 0.009), and arterial TN expression was elevated in CAD-positive patients (2.27% vs. 0.62%, *p* = 0.016). Multivariate analysis confirmed TN as an independent protective factor (OR = 0.680, 95% CI: 0.491–0.940, *p* = 0.020).

Similarly, in a proteomic analysis of diabetic patients, Rahim et al. [32] (*n* = 10, all male) observed significantly reduced TN levels in those with acute myocardial infarction compared to those without (0.908 ± 0.172 vs. 2.037 ± 0.321, *p* = 0.029), suggesting a potential involvement of TN in acute ischemic pathology.

Extending these findings to platelet proteomics, Maguire et al. [40] (*n* = 13) analyzed platelet releasates and found TN was uniquely present in patients with stable angina but absent in those with STEMI. Its absence, along with reduced levels of Factor V (F5) and fibronectin (FN1), defined a distinct pro-thrombotic platelet proteome in STEMI, potentially reflecting impaired fibrinolysis and altered clot resolution.

Supporting its broader relevance to cardiac dysfunction, Dixit et al. [33] (*n* = 13) conducted a prospective proteomic analysis in patients with coexisting atrial fibrillation, coronary microvascular dysfunction (CMD), and HF with preserved ejection fraction (HFpEF). Tetranectin was significantly upregulated in both ACH (AF + CMD + HFpEF) and CH (CMD + HFpEF) groups, with a fold change of 1.5. These findings suggest a potential compensatory or adaptive increase in TN expression in chronic cardiac dysfunction involving multiple pathophysiological mechanisms.

All findings described here are summarized in Table 4 below.

## 4. Discussion

This systematic review aimed to synthesize the current evidence on the prognostic significance of TN in HF and related cardiometabolic conditions. We sought to answer the following question: what is the role of TN, as a circulating or tissue-based biomarker, in predicting disease progression, clinical outcomes, and therapeutic response in patients with HF and cardiometabolic disorders?

### 4.1. Prognostic Value of Tetranectin in Heart Failure and Related Conditions

Five studies explicitly demonstrate the prognostic value of Tetranectin in predicting adverse cardiovascular outcomes across diverse clinical contexts.

Firstly, Ho et al. (2018) [34] with the 3523 patients, large cohort using a proteomic platform targeting 85 cardiovascular disease (CVD)-related proteins showing that higher TN levels were associated with a lower risk of incident HF (hazard ratio 0.82, *p* = 0.0067) and all-cause mortality, adjusted for clinical confounders.

Kopeva et al. (2023) [37] provided robust quantitative evidence in the context of anthracycline-related cardiac dysfunction (ARCD), reporting that lower baseline TN levels (≤15.9 ng/mL) independently predicted adverse outcomes over 24 months (odds ratio 7.08, 95% CI 2.26–15.85, *p* < 0.001), with a ROC AUC of 0.764. Notably, combining Tetranectin with NT-proBNP enhanced prognostic accuracy (AUC = 0.954, *p* = 0.002), highlighting its potential in risk stratification for ARCD.

Similarly, Shah et al. (2023) [35] showed consistent associations in HF patients, with higher Tetranectin levels linked to reduced risk of HF (HR = 0.82, *p* = 0.0063), all-cause mortality (HR = 0.82, *p* < 0.001), and cardiovascular death (HR = 0.77, *p* = 0.005).

Also, Patel-Murray et al. (2024) [36] focused on HFpEF in the PARAGON-HF trial, identifying Tetranectin among the top 10 proteins associated with lower risk of HF hospitalization and cardiovascular death (RR = 0.67 min. adj. *p* < 0.001; RR = 0.69 adjusted, *p* = 0.019).

Dib et al. (2024) [41] investigated HF patients and identified TN as one of the top proteins negatively associated with the composite endpoint of death or HF hospitalization (DHFA) and mortality alone, with findings replicated in the WashU HF registry. The consistent negative association with adverse outcomes in adjusted analyses underscores Tetranectin’s prognostic reliability in established HF.

The findings from Ho et al. (2018) [34], Dib et al. (2024) [41], and Kopeva et al. (2023) [37] collectively indicate that higher circulating Tetranectin levels are consistently associated with better outcomes across HF, ARCD, and mortality endpoints. Ho et al. (2018) [34] and Shah et al. (2023) [35] establish its protective role in broad CVD and HF populations, with consistent hazard ratios (HR = 0.82) for incident HF and mortality, and Shah et al. (2023) [35] further specifying cardiovascular death. Patel-Murray et al. (2024) [36] refine this in HFpEF, showing Tetranectin’s association with reduced HF hospitalization and cardiovascular death, with rate ratios (0.67–0.69) significant after risk factor adjustment. Dib et al. [41] (2024) reinforce this in established HF, with replication ensuring reliability.

Kopeva et al. (2023) [37] though previous findings, provide stronger statistical evidence by offering specific cutoff value and odds ratio in ARCD, alongside longitudinal data showing declining Tetranectin levels in patients with adverse outcomes, suggesting a dynamic prognostic role. The synergy with NT-proBNP observed by Kopeva et al. (2023) [37] strongly parallels the findings in afore mentioned contexts, suggesting that Tetranectin may enhance existing biomarker panels for improved risk prediction. These studies collectively support Tetranectin’s potential as a protective biomarker, with higher levels indicating a lower likelihood of adverse cardiovascular events.

Finally, a sixth study by McDonald et al. (2020) [38] indirectly supports Tetranectin’s prognostic potential by demonstrating reduced circulating levels in HF patients alongside elevated tissue expression linked to fibrotic genes (e.g., collagen I/III, MMPs). The inverse relationship shows that cardiac uptake may mitigate fibrosis, implicating Tetranectin in HF progression. Though not a primary focus, their findings align with above findings, reinforcing its biomarker potential for risk stratification. The observed link between lower circulating TN and adverse outcomes further highlights its diagnostic and predictive utility across HF and ASCVD.

It is worth noting that some discrepancies in findings may exist, as shown by a smaller cohort (*n* = 13) study by Dixit et al. [33] found no significant tetranectin changes in isolated HFpEF. Several explanations are plausible. Biologically, tetranectin is closely linked to extracellular matrix remodeling, collagen turnover, and fibrosis pathways (e.g., COL3A1, MMP9, TIMP1, galectin-3), with experimental data also suggesting anti-apoptotic and cardioprotective signaling via PI3K/Akt [38,41,43]. These mechanisms appear more pronounced in HFrEF and fibrosis-dominant HFpEF, whereas other HFpEF subgroups (e.g., obesity-/AF-driven, inflammation-dominant) may exhibit weaker or more variable TN signatures [44,45]. Methodologically, the Dixit et al. study [33] was underpowered, relied on a single time-point plasma measurement, and may have used narrower diagnostic criteria for HFpEF than other cohorts, all of which could reduce sensitivity to detect differences. Together, these factors show both the heterogeneity of HFpEF and the need for larger, well-phenotyped studies with standardized assays and integrated plasma–tissue analyses to clarify whether TN reliably distinguishes HFpEF subtypes.

Tetranectin is generally associated with better outcomes across HF, ARCD, and mortality contexts supports its potential for risk stratification, particularly when combined with other markers such as NT-proBNP. Further validation and mechanistic studies are needed to optimize its clinical application, while keeping cost-effectiveness in mind.

### 4.2. Diagnostic Accuracy

The diagnostic potential of Tetranectin (CLEC3B) in heart failure and related conditions, as evidenced by McDonald et al. (2020) [38], Dib et al. (2024) [41], and Vulciu et al. (2025) [43], suggests its utility in enhancing risk stratification and guiding personalized diagnostic approaches, though inconsistencies in specific HF subtypes warrant further exploration.

McDonald et al. (2020) [38] demonstrated that Tetranectin’s superior diagnostic accuracy over B-type natriuretic peptide (BNP) for HF, particularly when combined with BNP, could improve early detection in clinical settings, potentially allowing for timely intervention in patients with risk factors such as hypertension or diabetes.

Dib et al. (2024) [41] and Vulciu et al. (2025) [43] further support Tetranectin’s role in identifying HF, with consistent reductions in circulating levels across broad HF cohorts and in hypertensive/dyslipidemic patients with varying HF severity, respectively. These findings suggest that Tetranectin could complement existing biomarkers like BNP/NT-proBNP in diagnostic panels, enhancing specificity for HF diagnosis in heterogeneous populations.

However, Dixit et al. (2022) [33] found no significant changes in Tetranectin levels in HF with preserved ejection fraction (HFpEF), and Li et al. (2024) [42] reported upregulation in dilated cardiomyopathy (DCM), indicating that Tetranectin’s diagnostic utility may vary by HF subtype. These contextual dependencies, highlight the need for subtype-specific cutoffs and larger studies to validate Tetranectin’s role in nuanced diagnostic algorithms. Integrating Tetranectin into routine clinical diagnostics could facilitate earlier identification of at-risk patients, particularly when combined with echocardiographic parameters, as suggested by Vulciu et al. (2025) [43], but cost-effectiveness and standardization of assays remain critical considerations for widespread adoption.

Supplementary non-peer-reviewed findings (posters and preprints) further support Tetranectin’s diagnostic potential, offering complementary insights to the primary studies. Edgar et al. [46] reported a 30% reduction in Tetranectin levels in acute HF patients compared to breathless non-HF controls (*p* < 0.0001, AUC = 0.92), with earlier proteomic work identifying Tetranectin as a blood-based biomarker outperforming BNP, consistent with McDonald et al. (2020)’s [38] findings (AUC = 0.97 vs. 0.84). Their observation [47] of elevated Tetranectin in cardiac tissue of both HF with reduced ejection fraction (HFrEF) and HFpEF patients, correlating with multiple collagen subtypes (r = 0.31–0.78, *p* < 0.05), suggests a link to cardiac remodeling that could refine HF diagnosis by reflecting disease severity. Saha et al. [48] noted lower Tetranectin levels in DCM patients (*p* = 0.0006), achieving an AUC of 0.995 in a multi-marker model with lipids and β2-microglobulin, surpassing NT-proBNP alone (AUC = 0.965). These preliminary findings align with the diagnostic potential, suggesting that Tetranectin, particularly in multi-marker panels, could enhance diagnostic precision for acute HF and DCM.

Its ability to reflect HF severity could aid in early identification and risk stratification, but variability in expression across HF subtypes, as reported by Dixit et al. (2022) [33] and Li et al. (2024) [42], necessitates further research to establish subtype-specific thresholds and cost-effective assay standardization for clinical integration.

### 4.3. Mechanistic Insights and Animal Models

In the context of cardiovascular disease, experimental models have provided important clues about its protective role in ischemic injury and cardiac remodeling [49,50]. In vitro studies using hypoxic rat cardiomyocytes demonstrated that TN expression is downregulated under oxygen-deprivation conditions, while its overexpression increases cell viability, suppresses apoptosis, and activates the PI3K/Akt signaling pathway—crucial for cardiomyocyte survival [50]. These findings suggest that TN mitigates cellular damage during ischemia and may play a cardioprotective role via modulation of key intracellular pathways. To summarize the key roles and metabolic pathways revealed in this systematic review a visual display can be found in Figure 2.

Taking a brief look at animal models for possible different metabolic pathways, provides new insight. At models such as canine, it has been shown that the inverse pattern of TN expression is constant across. A study in which dogs with congestive HF were assessed, reported down-regulation of TN in stage D HF, aligning with earlier findings in both canine and human cardiovascular disease [26,32,51,52]. Interestingly, low TN levels in dogs were more closely associated with CHF than B-type natriuretic peptide, suggesting that TN may also serve as a reliable biomarker candidate for HF in veterinary cardiology [38].

Further support comes from single-cell transcriptomic analyses of myocardial tissue, where CLEC3B expression correlated with lower fibrosis and improved metabolic resilience in dilated cardiomyopathy and peripartum cardiomyopathy [42]. Interestingly, TN has also been implicated in longevity-associated pathways and cancer-related extracellular matrix regulation, suggesting its broader involvement in systemic homeostasis and repair mechanisms [53,54,55].

Although the primary focus remains on cardiovascular conditions, similar anti-apoptotic functions of tetranectin have been observed in neurodegenerative disease. In a Parkinson’s disease model, tetranectin protected dopaminergic neurons from mitochondrial toxin-induced apoptosis and improved motor outcomes via modulation of the p53/Bax pathway [23].

These findings support the hypothesis that tetranectin may act as a general stress-response protein, preserving cellular integrity across organ systems, including the heart.

### 4.4. Context-Dependent Roles in Cardiometabolic Diseases

Tetranectin demonstrates differential expression across a spectrum of cardiometabolic conditions, suggesting a pathophysiological specific role in cardiovascular remodeling and metabolic stress responses. In a longitudinal cohort of anthracycline-treated breast cancer patients without prior cardiovascular disease, declining serum tetranectin levels over 24 months were independently associated with adverse outcomes in anthracycline-related cardiac dysfunction (ARCD), outperforming NT-proBNP in prognostic accuracy [37]. The combined use of both biomarkers further enhanced predictive performance, indicating complementary mechanistic pathways.

Proteomic analyses of myocardial tissue in peripartum cardiomyopathy [42] and dilated cardiomyopathy [48] have shown reduced tetranectin levels, supporting its involvement in myocardial structural remodeling and fibrosis. Moreover, in patients with type 2 diabetes mellitus, tetranectin levels were significantly decreased following acute myocardial infarction, highlighting a potential role in acute ischemic injury and impaired reparative processes [32].

Collectively, these findings indicate that tetranectin expression is not uniformly altered across cardiovascular conditions but instead reflects disease-specific pathophysiological processes, supporting its potential utility as a context-sensitive biomarker in cardiometabolic disease stratification.

### 4.5. Proposed Cut-Off Values and Threshold Ranges for Tetranectin

Several studies in this review identified clinically relevant cut-off values or concentration ranges for TN, which may aid in risk stratification and disease discrimination across cardiovascular conditions.

In anthracycline-related cardiac dysfunction, Kopeva et al. [37] identified a serum tetranectin cut-off value of ≤15.9 ng/mL as a strong predictor of adverse outcomes over a 24-month follow-up. Patients with ARCD and a progressive clinical course showed significantly lower TN levels (median 13.25 ng/mL [IQR: 9.55–16.5]) compared to those with a stable disease course (17.9 ng/mL [15.8–20.8], *p* < 0.001). The prognostic accuracy of this cut-off was further enhanced by combining TN with NT-proBNP, achieving an AUC of 0.954 (*p* = 0.002).

In a preprint regarding dilated cardiomyopathy (DCM), Saha et al. [48] reported that tetranectin levels were significantly lower in DCM patients compared to controls (1.99 ± 0.88 μg/mL vs. 2.49 ± 0.90 μg/mL, *p* = 0.0006). When integrated into a multi-marker model with lipids and β2-microglobulin, the diagnostic performance reached an AUC of 0.995, outperforming NT-proBNP alone (AUC = 0.965).

In coronary artery disease (CAD), Chen et al. [26] described a decrease in serum TN levels corresponding to disease severity. TN concentrations declined from 11.11 mg/mL in CAD-negative individuals to 9.30 mg/mL in those with three-vessel disease (*p* for trend = 0.009), supporting the use of dynamic thresholds in stratifying atherosclerotic burden.

In type 2 diabetes mellitus with acute myocardial infarction (AMI), Rahim et al. [32] found significantly lower TN values in AMI patients (0.908 vs. 2.037 normalized volume units, *p* = 0.029), corresponding to a −2.2 fold change. Although a numerical cut-off was not established, the contrast between groups was valid.

In hypertensive patients with HF, Vulciu et al. [43] observed declining TN levels across HF severity strata: from 17.92 mg/L in controls to 5.34 mg/L in moderate HF and 2.70 mg/L in severe HF (*p* < 0.01), suggesting that levels below ~5 mg/L may indicate advanced disease.

While ELISA assays are the most widely used and clinically practical, proteomic platforms such as LC–MS/MS and SWATH-MS offer greater sensitivity and specificity, enabling detection of low-abundance proteins and multi-marker profiling. Aptamer-based platforms such as SomaScan achieve femtomolar sensitivity, though results are expressed as relative rather than absolute concentrations and require further clinical validation. Consequently, any proposed cut-offs should be considered platform-specific and exploratory until cross-platform calibration and standardized assays are established.

An overview of the different measurement methods and units reported across discussed studies in Section 4.5 is provided in Table 5.

To summarise, these findings suggest that tetranectin thresholds vary depending on the population, disease stage, and assay method, but low circulating levels consistently associate with worse outcomes. The proposed cut-offs—ranging from ~2–15 mg/L or ng/mL—could serve as starting points for clinical risk models once standardized assays are adopted.

### 4.6. Emerging Pediatric and Developmental Cardiovascular Contexts

Although most studies on tetranectin have focused on adult cardiometabolic and HF phenotypes, emerging evidence suggests potential relevance in pediatric cardiovascular diseases.

In a proteomic analysis [56] of myocardial tissue from Kawasaki disease patients, tetranectin (P05452) was identified with 42% sequence coverage and a high protein score (295), indicating significant expression changes during acute vasculitis and coronary artery involvement. Given Kawasaki disease’s association with vascular inflammation and remodeling, altered TN levels may reflect pathophysiological processes similar to those seen in adult HF, though through distinct immunoinflammatory mechanisms.

Furthermore, a genomic study by Ping Li et al. [57], in congenital heart disease (CHD) has identified CLEC3B among differentially expressed genes in syndromic CHD cases, particularly those involving atrial and ventricular septal defects, as well as abnormal cardiac morphology. Its presence among disease-associated genes (OMIM-linked) in the DECIPHER database cohort shows a possible developmental role in cardiogenesis and structural heart defects.

These pediatric insights, though preliminary, support the hypothesis that CLEC3B may serve not only as a context-dependent biomarker in adults’ conditions but also as a molecular participant in early-life cardiac pathology. Further investigations are warranted to clarify its mechanistic roles and potential utility in pediatric cardiovascular risk stratification.

### 4.7. Clinical Implications and Future Directions

Tetranectin has shown substantial potential as a biomarker in cardiovascular diseases, particularly HF and cardiometabolic conditions. Its consistent association with adverse remodeling, reduced survival, and additive diagnostic value—especially when combined with established markers such as NT-proBNP—suggests that tetranectin could enhance current risk stratification strategies. In addition to tetranectin, several established biomarkers provide diagnostic and prognostic value in cardiovascular disease. Von Willebrand factor (vWF), a marker of endothelial dysfunction and thrombo-inflammation, has been consistently linked to coronary artery disease, heart failure, and stroke, as well as adverse outcomes across diverse cohorts [58]. While vWF highlights vascular injury pathways, tetranectin reflects extracellular matrix remodeling and fibrosis, underscoring the value of complementary multi-pathway biomarker approaches. Furthermore, tissue-level correlations with fibrotic pathways reinforce its mechanistic relevance in disease progression, highlighting its utility beyond passive risk association.

Despite these promising findings, several steps are required before tetranectin can be adopted in clinical practice. Future research should focus on standardizing quantification methods across platforms, ensuring analytical reproducibility and interlaboratory consistency. Establishing validated disease-specific cut-off values in large, diverse populations will be essential for diagnostic accuracy and prognostic reliability. Moreover, integration of tetranectin into existing multi-marker algorithms should be tested to evaluate whether it provides incremental value over current models. Longitudinal studies assessing dynamic changes in circulating tetranectin levels in response to therapy or disease progression could provide insights into its utility for treatment monitoring. In parallel, molecular and cellular investigations are needed to elucidate role of TN and other involved molecules in processes such as myocardial fibrosis, extracellular matrix remodeling, and immune-metabolic signaling.

Importantly, tetranectin may prove especially valuable in specific patient subgroups. In fibrosis-dominant HFpEF, it could help identify patients at greatest risk of progression, complementing echocardiographic indices of diastolic dysfunction. In anthracycline-related cardiotoxicity, tetranectin may allow earlier detection of myocardial injury compared with natriuretic peptides or troponin, enabling timely cardioprotective interventions. In chronic HFrEF and high-risk cohorts, tetranectin could add incremental prognostic value to existing biomarker panels, informing closer monitoring or treatment escalation. Such subgroup-specific applications illustrate the potential for tetranectin to refine diagnostic and prognostic algorithms and support more personalized management of HF and cardiometabolic disease.

Taken together, the evidence reviewed herein suggests that tetranectin has the potential to serve as both a prognostic and diagnostic biomarker, particularly in HF, CAD, and cardiotoxicity monitoring. Its consistent association with major cardiovascular outcomes and its strong biological links to myocardial fibrosis underscore the promise of CLEC3B as a clinically relevant marker that, once validated, could be integrated into routine cardiovascular care.

### 4.8. Limitations

This systematic review, while comprehensive, is subject to several limitations. The evidence base is small and heterogeneous, with predominantly observational designs (cross-sectional or retrospective), diverse populations, variable disease definitions and endpoints, and widely ranging sample sizes—including small cohorts—limiting statistical power, causal inference, and generalizability. Tetranectin quantification was not standardized (assay type, units, reference ranges), few studies reported validated cut-off thresholds, and statistical adjustment for key confounders (e.g., comorbidities, medication use) was often incomplete, increasing the risk of bias and overestimation of associations. Measurement approaches varied (e.g., ELISA vs. proteomics), reporting quality was uneven, sex-stratified or subgroup analyses were uncommon, and external validation was scarce. Moreover, many studies focused on specific populations (e.g., HFpEF, chemotherapy-induced cardiomyopathy, Asian cohorts), which may not reflect broader clinical settings. At the review level, the absence of a formal meta-analysis precluded quantitative synthesis and formal assessment of between-study heterogeneity, and despite a systematic search strategy, relevant studies may have been missed due to database coverage or language restrictions. As the field evolves, larger, prospective, and methodologically standardized investigations are needed to establish the clinical validity and utility of tetranectin as a cardiovascular biomarker.

## 5. Conclusions

This systematic review provides evidence for a significant association between Tetranectin and the pathogenesis of heart failure (HF) and related cardiometabolic conditions. Our study reveals that patients with HF, including those with heart failure with preserved ejection fraction (HFpEF), heart failure with reduced ejection fraction (HFrEF), and anthracycline-related cardiac dysfunction (ARCD), often exhibit reduced circulating Tetranectin levels, alongside elevated cardiac tissue expression associated with fibrosis.

Studies included demonstrate that higher Tetranectin levels are consistently linked to a lower risk of adverse outcomes, such as incident HF, hospitalization, and mortality, with hazard ratios ranging from 0.67 to 0.82 across diverse and substantial cohorts. Experimental models further suggest that Tetranectin exerts cardioprotective effects by enhancing cardiomyocyte survival through the PI3K/Akt pathway and mitigating fibrosis-related remodeling. These effects are likely mediated through anti-apoptotic, anti-fibrotic, and tissue remodeling mechanisms, involving pathways such as plasminogen binding and extracellular matrix regulation.

Translation will require standardized, validated assays (harmonized units, reference materials, prespecified cut-offs); prospective multicenter cohorts with rigorous confounder control, external validation, and diverse, sex-stratified samples; head-to-head and incremental-value analyses versus NT-proBNP/hs-troponin; longitudinal studies of trajectories and treatment response; and interventional evidence, including randomized trials of Tetranectin-guided care plus implementation and cost-effectiveness evaluations.

Considered together, these findings show the potential of Tetranectin as a prognostic and diagnostic biomarker in HF, suggesting that strategies to modulate its expression or activity may offer new possibilities for risk stratification and therapeutic intervention in HF and related cardiometabolic disorders.

## Figures and Tables

**Table 1:** Overview table of the selected studies. TN—Serum tetranectin; CAD—Coronary artery disease; CVD—Cardiovascular disease; ASCVD—Atherosclerotic cardiovascular disease; AMI—Acute myocardial infarction; HF—Heart failure; ACH—Study-specific phenogroup: atrial fibrillation (A), coronary microvascular disease (C), heart failure with preserved ejection fraction (H); CH—Study-specific phenogroup: coronary microvascular disease (C) and heart failure with preserved ejection fraction (H); CMD—Coronary microvascular disease; HFpEF—Heart failure with preserved ejection fraction; HFrEF—Heart failure with reduced ejection fraction; ARCD—Anthracycline-related cardiac dysfunction; DHFA—Death or heart failure–related hospital admission; LVEF—Left ventricular ejection fraction; LC–MS/MS—Liquid chromatography–tandem mass spectrometry; DCM—Dilated cardiomyopathy; NA—Not Applicable.

Author	Year	Country	Study Design	Number of Patients ^1^	Number of Male Patients ^1^	Mean/Median Age ^2^	Number of Control Patients	Tetranectin Measurement	Assessed Outcomes	Endpoints Reported
Chen [26]	2015	China	Cross-sectional	316	238	66 ± 10	96	ELISA kit	Tetranectin levels, correlation with lipid profile and inflammatory markers	TN levels are independently associated with stable CAD, with decreased levels in patients linked to increased uptake by atherosclerotic plaques, as evidenced by high TN expression in lesions and greater reductions in three-vessel disease.
Ho [34]	2018	USA	Prospective Cohort	3523	1644	62 ± 8	NA	ELISA kit	ASCVD, coronary heart disease death, HF, all-cause mortality, with a secondary end point of CVD-related death	TN shows protective associations with HF, all-cause mortality, and CVD death, suggesting it may serve as a potential prognostic biomarker. However, its inclusion in predictive models was less frequent compared to more robust biomarkers like NT-proBNP or GDF15, indicating that its prognostic power may be context-dependent or complementary.
Rahim [32]	2018	Malaysia	Cross-sectional	10	10	56.7 ± 8.4	10	ELISA kit	Plasma protein expression levels	Tetranectin levels were significantly lower in T2DM patients with AMI compared to those without AMI, suggesting tetranectin as a potential biomarker for AMI in T2DM patients.
McDonald [38]	2020	Ireland	Retrospective and Prospective Cohort	40	NA	72 ± 13	20	ELISA kit	Correlation of TN levels with fibrosis and inflammation. Stratification by TN levels, diagnostic performance.	Tetranectin levels are significantly lower in HF patients and outperform the standard biomarker BNP in diagnostic specificity and sensitivity for HF. Combining Tetranectin with BNP enhances HF diagnosis accuracy, unaffected by age or gender. While serum Tetranectin levels negatively correlate with circulating fibrosis markers TN is a promising candidate HF biomarker associated with fibrotic processes within the myocardium.
Maguire [40]	2020	Germany	Retrospective Cohort	13	11	71.46 ± 11.8	14	MS and MS/MS	Differential protein release in platelet release between STEMI and SAP	Tetranectin was uniquely present in the platelet releasate of stable angina patients and absent in STEMI patients. Its absence, along with reduced levels of coagulation factor V and fibronectin, defines a moderated proteomic network associated with acute myocardial infarction. TN is linked to extracellular vesicles and plasminogen activation, suggesting its depletion may reflect impaired fibrinolytic activity in STEMI.
Dixit [33]	2022	USA	Prospective Cohort	13	NA	57 ^2^	5	SomaScan assay	Plasma protein expression levels	TN was found to be upregulated in patients with AF, CMD, and HFpEF (ACH and CH groups), while it was downregulated in isolated CMD. Potential use as differential biomarker across HF subtypes.
Kopeva [37]	2023	Russian Federation	Retrospective Case-Control	114	0	48 ^2^	248	ELISA kit	Adverse course of ARCD	TN showed predictive value for adverse outcomes in ARCD, and its diagnostic accuracy significantly improved when combined with NT-proBNP, suggesting potential utility as a complementary biomarker in cardiovascular risk stratification.
Dib [41]	2024	USA	Prospective Cohort	1167	798	56.8 ± 0.9	1067	SomaScan assay	All-cause mortality and DHFA in HF	Higher levels of TN were associated with a lower risk of death and DHFA.
Li [42]	2024	Australia	Retrospective Cohort	12	0	36.9 ± 10.3	18	LC-MS/MS	Plasma protein expression levels	TN is upregulated in DCM.
Patel- Murray [36]	2024	USA	Prospective Cohort	1117	539	73.5 ± 8.0	NA	SomaScan assay	Plasma protein expression levels	Higher levels of TN were significantly associated with a reduced risk of HF hospitalization and cardiovascular death in HFpEF patients, suggesting its potential utility as a protective prognostic biomarker.
Shah [35]	2024	USA and Norway	Prospective Cohort	18,383	8722	64.6 ± 6.7	NA	SomaScan assay	Plasma protein expression levels	TN was consistently associated with a lower risk of HF across all three cohorts (Visit 5, Visit 3, and HUNT). Its replication across independent datasets highlights its potential role as a protective biomarker in HF risk stratification.
Vulciu [43]	2025	Romania	Cross-sectional	67	33	56.56 ± 13.82	20	ELISA kit	Relationship between tetranectin levels and the severity of HF in patients with hypertension and dyslipidemia	TN levels decrease progressively with worsening HF (NYHA class II–IV). TN positively associated with LVEF; negatively associated with diastolic dysfunction.

^1^ Number of patients with cardiac/vascular disease; ^2^ Mean age of patients with cardiac/vascular disease.

**Table 2:** Tetranectin as a Prognostic Biomarker for Heart Failure and Related Outcomes. ASCVD—Atherosclerotic cardiovascular disease; HF—Heart failure; CVD—Cardiovascular disease; ARCD—Anthracycline-related cardiac dysfunction; HFpEF—Heart failure with preserved ejection fraction; DHFA—Death or heart failure—related hospital admission.

Author	Assessed Outcomes	Key Findings
Ho [34]	ASCVD, coronary heart disease death, HF, all-cause mortality, with a secondary end point of CVD-related death	Higher plasma levels of TN were significantly associated with a reduced risk of HF (HR: 0.82; 95% CI: 0.71–0.95; *p* = 6.3 × 10^−3^), all-cause mortality (HR: 0.82; 95% CI: 0.76–0.88; *p* = 2.9 × 10^−7^) and cardiovascular death (HR: 0.77; 95% CI: 0.64–0.92; *p* = 5.1 × 10^−3^)
Kopeva [37]	Adverse course of ARCD	In patients with an adverse course of ARCD, serum TN levels were significantly lower—by 27.6% compared to those with favorable ARCD and 33.7% compared to those without ARCD (*p* < 0.001). Tetranectin levels decreased significantly over 24 months in this group (from 11.8 to 9.02 ng/mL, *p* < 0.001), while they remained stable in other groups. Importantly, TN emerged as an independent predictor of adverse ARCD outcomes (OR = 7.08, *p* < 0.001), with a cut-off value ≤ 15.9 ng/mL showing predictive power (AUC = 0.764, *p* < 0.001). Although NT-proBNP alone lacked prognostic value, its combination with TN greatly improved predictive accuracy (AUC = 0.954, *p* = 0.002).
Patel- Murray [36]	Plasma protein expression levels	In patients with HFpEF, higher circulating levels of TN were significantly associated with a lower risk of HF hospitalization and cardiovascular death, with a minimally adjusted rate ratio of 0.67 (95% CI: 0.56–0.81, *p* = 4.0 × 10^−5^) and a risk factor–adjusted rate ratio of 0.69 (95% CI: 0.51–0.94, *p* = 0.019).
Dib [41]	All-cause mortality and DHFA in HF	TN is one of the top proteins negatively associated with the composite endpoint of death or HF hospitalization (DHFA) and mortality alone, with findings replicated in the WashU HF registry.
Shah [35]	Plasma protein expression levels	TN is consistently associated with a lower risk of HF (HR = 0.82, 95% CI: 0.71–0.95, *p* = 0.0063), all-cause mortality (HR = 0.82, 95% CI: 0.76–0.88, *p* = 2.9 × 10^−7^), and cardiovascular death (HR = 0.77, 95% CI: 0.64–0.92, *p* = 0.0051). In HFpEF patients, it was among the top proteins linked to reduced hospitalization and CV death (RR = 0.67 minimally adjusted, *p* = 4 × 10^−5^; RR = 0.69 adjusted, *p* = 0.019).

**Table 3:** Diagnostic Utility of Tetranectin in Heart Failure. TN—Serum tetranectin; HF—Heart Failure; DCM—Dilatative Cardiomyopathy.

Author	Assessed Outcomes	Key Findings
McDonald [38]	Correlation of TN levels with fibrosis and inflammation. Stratification by TN levels, diagnostic performance.	TN levels were significantly reduced in HF patients (*p* < 0.0001) and were more strongly associated with HF than B-type natriuretic peptide (AUC 0.97 vs. 0.84, *p* = 0.011). Cardiac tissue expression of TN showed positive correlations with fibrotic genes: COL3A1 (r = 0.37, *p* = 0.036), MMP9 (r = 0.49, *p* = 0.005), TIMP1 (r = 0.41, *p* = 0.019), and galectin-3 (r = 0.59, *p* = 0.0004). TN protein levels in tissue also correlated positively with collagen content (r = 0.55, *p* = 0.0019) and were higher in samples with increased fibrosis (*p* = 0.011).
Dixit [33]	Plasma protein expression levels	TN has not significantly changed in HFpEF alone (*p* = 0.9).
Dib [41]	All-cause mortality and DHFA in HF	TN was found to be significantly downregulated in the plasma of HF patients compared to controls (*p* < 0.001).
Li [42]	Plasma protein expression levels	In DCM, TN was significantly upregulated compared to donor hearts (FC = 3.1, PBH = 2.0 × 10^−4^).
Vulciu [43]	Relationship between TN levels and the severity of HF in patients with hypertension and dyslipidemia	TN emerged as a significant independent predictor. Higher serum TN levels were associated with lower HF severity, with an OR of 0.998 per 1 mg/L increase (95% CI: 0.997–0.999, *p* = 0.002), after adjusting for age, diabetes, sex, LVEF, and PON1.

**Table 4:** Tetranectin Expression and Associations with Disease Severity in CAD and Cardiometabolic Cohorts. TN—Serum tetranectin; CAD—Coronary artery disease; ACH—Study-specific phenogroup: atrial fibrillation (A), coronary microvascular disease (C), heart failure with preserved ejection fraction (H); CH—Study-specific phenogroup: coronary microvascular disease (C) and heart failure with preserved ejection fraction (H); CMD—Coronary microvascular disease; AMI—Acute myocardial infarction.

Author	Assessed Outcomes	Key Findings
Chen [26]	TN levels, correlation with lipid profile and inflammatory markers	In CAD patients, serum TN levels were significantly lower than in healthy controls (10.12 ± 3.41 mg/mL vs. 11.16 ± 3.17 mg/mL, *p* = 0.007). TN levels declined with disease severity: 11.11 mg/mL in CAD-negative, 10.41 mg/mL in one-vessel, 9.90 mg/mL in two-vessel, and 9.30 mg/mL in three-vessel disease (*p* for trend = 0.009). Significant differences were found between CAD-negative and two-vessel (*p* = 0.014) and three-vessel disease (*p* < 0.001), and between one- and three-vessel disease (*p* = 0.018). Arterial TN expression was higher in CAD-positive vs. controls (2.27% vs. 0.62%, *p* = 0.016). Multivariate analysis confirmed TN as an independent predictor of CAD (OR = 0.680, 95% CI: 0.491–0.940, *p* = 0.020).
Rahim [32]	Plasma protein expression levels	In T2DM patients, TN levels were significantly lower in those with AMI compared to those without AMI (0.908 ± 0.172 vs. 2.037 ± 0.321, *p* = 0.029), showing a fold change of −2.2.
Maguire [40]	Differential protein release in platelet release between STEMI and SAP	TN was uniquely present in stable angina patients and absent in STEMI. TN is involved in plasminogen activation and may reflect intact fibrinolysis. Its absence, along with decreased F5 and FN1, defines a moderated platelet protein network in STEMI, possibly reflecting altered platelet activation and impaired clot resolution. Prior evidence links CLEC3B to CAD progression, highlighting its potential as a non-invasive marker.
Dixit [33]	Plasma protein expression levels	TN is upregulated (fold change = 1.5) in both ACH (atrial fibrillation + CMD + HFpEF) and CH (CMD + HFpEF).

**Table 5:** Measurement methods and reported units of tetranectin across studies. TN—Tetranectin; ELISA—Enzyme-linked immunosorbent assay; LC–MS/MS—Liquid chromatography–tandem mass spectrometry; SWATH-MS—Sequential window acquisition of all theoretical mass spectra; DCM—Dilated cardiomyopathy; ARCD—Anthracycline-related cardiac dysfunction; CAD—Coronary artery disease; AMI—Acute myocardial infarction; HF—Heart failure.

Study	Condition	Assay/Platform	Units of Measure Reported
Chen [26]	CAD	ELISA (MyBioSource)	mg/mL
Rahim [32]	T2DM + AMI	2D gel + ELISA validation	Normalized volume units
Kopeva [37]	ARCD	ELISA (RayBio)	ng/mL
Saha [48]	DCM	SWATH-MS proteomics + ELISA validation	μg/mL/relative intensity
Vulciu [43]	HF	ELISA (MyBioSource)	ng/mL

## Data Availability

The original contributions presented in this study are included in the article. Further inquiries can be directed to the corresponding author.

## Supplementary Materials

The following supporting information can be downloaded at: https://www.mdpi.com/article/10.3390/medsci13040206/s1, Table S1: Quality Assessment of Included Studies.

## Abbreviations

The following abbreviations are used in this manuscript:

ACH	Study-specific phenogroup: atrial fibrillation (A), coronary microvascular disease (C), heart failure with preserved ejection fraction (H)
AMI	Acute Myocardial Infarction
ARCD	Anthracycline related cardiac dysfunction
ASCVD	Atherosclerotic cardiovascular disease
CAD	Coronary artery disease
CH	Coronary microvascular disease & heart failure with preserved ejection fraction
CHD	Congenital heart disease
CMD	Coronary microvascular disease
DCM	Dilated cardiomyopathy
DHFA	Death or heart failure–related hospital admission
HF	Heart failure
HFpEF	Heart failure with preserved ejection fraction
HFrEF	Heart failure with reduced ejection fraction
LC–MS/MS	Liquid chromatography–tandem mass spectrometry
LVEF	Left Ventricular Ejection Fraction
vWF	Von Willebrand factor
TN	Tetranectin
