# Supplementary material for: Diagnostic and Prognostic Potential of Tetranectin in Heart Failure and Cardiovascular Disease: A Systematic Review
Source: Med Sci (Basel). 2025 Sep 24;13(4):206. doi: 10.3390/medsci13040206 (PMC12550903; doi:10.3390/medsci13040206)
Supplement: Supplementary file 1 [file medsci-13-00206-s001.zip › medsci-3839129-supplementary.pdf]

medical sciences

MDPI

Systematic Review

Diagnostic and Prognostic Potential of Tetranectin in Heart Failure and Cardiovascular Disease: A Systematic Review

Supplemental Table S1. Quality Assessment of Included Studies. Y = yes; N = no; CD = cannot determine; NA = not applicable; NR = not reported; CR= Cross-sectional; ROC=Retrospective Cohort Study; PCS=Prospective Cohort Study; RCC= Retrospective Case-Control.

**Author**	**Study Design**	1	2	3	4	5	6	7	8	9	10	11	12	13	14	Quality
Chen[26]	CR	Y	Y	Y	Y	N	Y	CD	Y	Y	N	Y	Y	NA	Y	Good
Ho[34]	PCS	Y	Y	Y	Y	N	Y	Y	Y	Y	N	Y	Y	Y	Y	Good
Rahim[32]	CR	Y	Y	Y	Y	N	Y	CD	N	Y	N	Y	Y	NA	N	Fair
McDonald[38]	PCS	Y	Y	NR	Y	N	Y	Y	Y	Y	N	Y	Y	NA	Y	Good
Maguire[40]	ROC	Y	Y	NR	Y	N	Y	Y	Y	Y	N	Y	CD	NR	Y	Good
Dixit[33]	PCS	Y	Y	NR	Y	N	Y	CD	Y	Y	N	Y	Y	NA	Y	Good
Kopeva[37]	RCC	Y	Y	NR	Y	N	Y	Y	Y	Y	Y	Y	Y	Y	Y	Good
Dib[41]	PCS	Y	Y	NR	Y	N	Y	Y	Y	Y	N	Y	Y	Y	Y	Good
Li[42]	RCS	Y	Y	NR	Y	N	Y	CD	Y	Y	N	Y	Y	NA	N	Fair
Patel-Murray [36]	PCS	Y	Y	NR	Y	N	Y	Y	Y	Y	N	Y	Y	Y	Y	Good
Shah[35]	PCS	Y	Y	NR	Y	Y	Y	Y	Y	Y	N	Y	Y	Y	Y	Good
Vulciu[43]	CR	Y	Y	NR	Y	N	Y	Y	Y	Y	N	Y	Y	Y	Y	Good

 https://doi.org/10.3390/medsci13040206
